# The diagnostic accuracy of inferior vena cava respiratory variation in predicting volume responsiveness in patients under different breathing status following abdominal surgery

**DOI:** 10.1186/s12871-022-01598-5

**Published:** 2022-03-08

**Authors:** Qian Ma, Xueduo Shi, Jingjing Ji, Luning Chen, Yali Tian, Jing Hao, Bingbing Li

**Affiliations:** Department of Anesthesiology, Nanjing Drum Tower Hospital, The Affiliated Hosptial of Nanjing University Medical School, 321 Zhongshan Road, 210008 Nanjing, China

**Keywords:** Abdominal surgery, Volume responsiveness, Inferior vena cava, Respiratory variation, Ultrasound

## Abstract

**Background:**

The validation of inferior vena cava (IVC) respiratory variation for predicting volume responsiveness is still under debate, especially in spontaneously breathing patients. The present study aims to verify the effectiveness and accuracy of IVC variability for volume assessment in the patients after abdominal surgery under artificially or spontaneously breathing.

**Methods:**

A total of fifty-six patients after abdominal surgeries in the anesthesia intensive care unit ward were included. All patients received ultrasonographic examination before and after the fluid challenge of 5 ml/kg crystalloid within 15 min. The same measurements were performed when the patients were extubated. The IVC diameter, blood flow velocity–time integral of the left ventricular outflow tract, and cardiac output (CO) were recorded. Responders were defined as an increment in CO of 15% or more from baseline.

**Results:**

There were 33 (58.9%) mechanically ventilated patients and 22 (39.3%) spontaneously breathing patients responding to fluid resuscitation, respectively. The area under the curve was 0.80 (95% CI: 0.68–0.90) for the IVC dimeter variation (cIVC1) in mechanically ventilated patients, 0.87 (95% CI: 0.75–0.94) for the collapsibility of IVC (cIVC2), and 0.85 (95% CI: 0.73–0.93) for the minimum IVC diameter (IVCmin) in spontaneously breathing patients. The optimal cutoff value was 15.32% for cIVC1, 30.25% for cIVC2, and 1.14 cm for IVCmin. Furthermore, the gray zone for cIVC2 was 30.72 to 38.32% and included 23.2% of spontaneously breathing patients, while 17.01 to 25.93% for cIVC1 comprising 44.6% of mechanically ventilated patients. Multivariable logistic regression analysis indicated that cIVC was an independent predictor of volume assessment for patients after surgery irrespective of breathing modes.

**Conclusion:**

IVC respiratory variation is validated in predicting patients' volume responsiveness after abdominal surgery irrespective of the respiratory modes. However, cIVC or IVCmin in spontaneously breathing patients was superior to cIVC in mechanically ventilated patients in terms of clinical utility, with few subjects in the gray zone for the volume responsiveness appraisal.

**Trial registration:**

ChiCTR-INR-17013093. Initial registration date was 24/10/2017.

**Supplementary Information:**

The online version contains supplementary material available at 10.1186/s12871-022-01598-5.

## Background

The purpose of fluid therapy is to attain the maximal increase in cardiac output (CO) after fluid replenishment [1]. However, nearly half of patients couldn’t increase CO following volume expansion, which, on the contrary, can be eventually complicated with cardiopulmonary dysfunction due to fluid overload [2, 3] Thus, the goal-directed fluid therapy based on the volume status assessment played a pivotal role in the enhanced recovery for surgical patients.

Currently, conventional static indicators comprising central venous pressure (CVP), urine output, et al., are poorly associated with volume status and cannot accurately predict patients' volume responsiveness [4]. In addition, pulse pressure variation (PPV) [5] or stroke volume variation (SVV) [6] from dynamic indicators, only candidates for a particular specific population with the regular heart rhythm under mechanical ventilation mode [7]. However, IVC ultrasonography, as a non-invasive, reproducible, real-time examination tool, has gained increasing popularity in volume responsiveness appraisal. A growing number of researches have demonstrated the significance of IVC variation indices for assessing volume responsiveness in mechanically ventilated patients suffering from septic shock or circulatory instability in the intensive care unit (ICU) [8]. However, the study conducted by de Oliveira [9] suggested that the individual PPV discriminative properties for predicting volume responsiveness in postoperative mechanically ventilated patients seemed superior to those parameters from IVC respiratory variability. Moreover, the consensus on the effectiveness of IVC variation indices to predict volume responsiveness hasn’t been reached simultaneously in spontaneously breathing patients due to the uncontrolled or variable intrathoracic pressure. A large prospective study indicated that IVC collapsibility performed well in predicting volume responsiveness in spontaneously breathing critically-ill patients, with an AUC of 0.84 (0.76, 0.91) [10]. However, the study conducted by Airapetian N demonstrated that neither IVC diameter nor IVC collapsibility index was predictive of volume responsiveness in the spontaneously breathing patients hospitalized in ICU, unless the collapsibility index of 42% or more was utilized [11]. Furthermore, a meta-analysis [12] including 17 studies consisting of 533 patients studied the reliability of IVC variation index in patients, and the results showed that the area under the receiver operating characteristic (AUC) for IVC variability ranged from 0.31 to 0.91. The authors concluded that the parameters derived from IVC dimension was of little clinical value, especially in spontaneously breathing patients.

Therefore, the present study aimed to evaluate the validity and accuracy of respiratory variation in IVC diameter to predict the volume responsiveness in patients after surgery under artificial or spontaneous breathing mode and provided a new train of thought for fluid management.

## Methods

### Ethics approval and consent to participate

This study was approved by the ethics committee of Nanjing Drum Tower Hospital with approval number [No.2017–122-02]. The patient provided written consent. All methods were carried out in accordance with Declaration of Helsinki.

### Participants

From May 2018 to December 2018, a total of 60 consecutive patients scheduled for elective major abdominal operation in the anesthesia ICU (AICU) were recruited in this study. The inclusion criteria comprise the patients aged between 20 to 70-year-old, with American Society of Anesthesiologists (ASA) physical status classification I to III, body mass index (BMI) with the range of 20–26 kg/m^2^. The patients with an irregular rhythm, cardiopulmonary dysfunction, liver, kidney function failure, incomplete clinical data, difficulty in delineating the anatomic structure due to obscure images of IVC and heart by transthoracic ultrasound examination were excluded.

### Study design

**Fig. 1:**
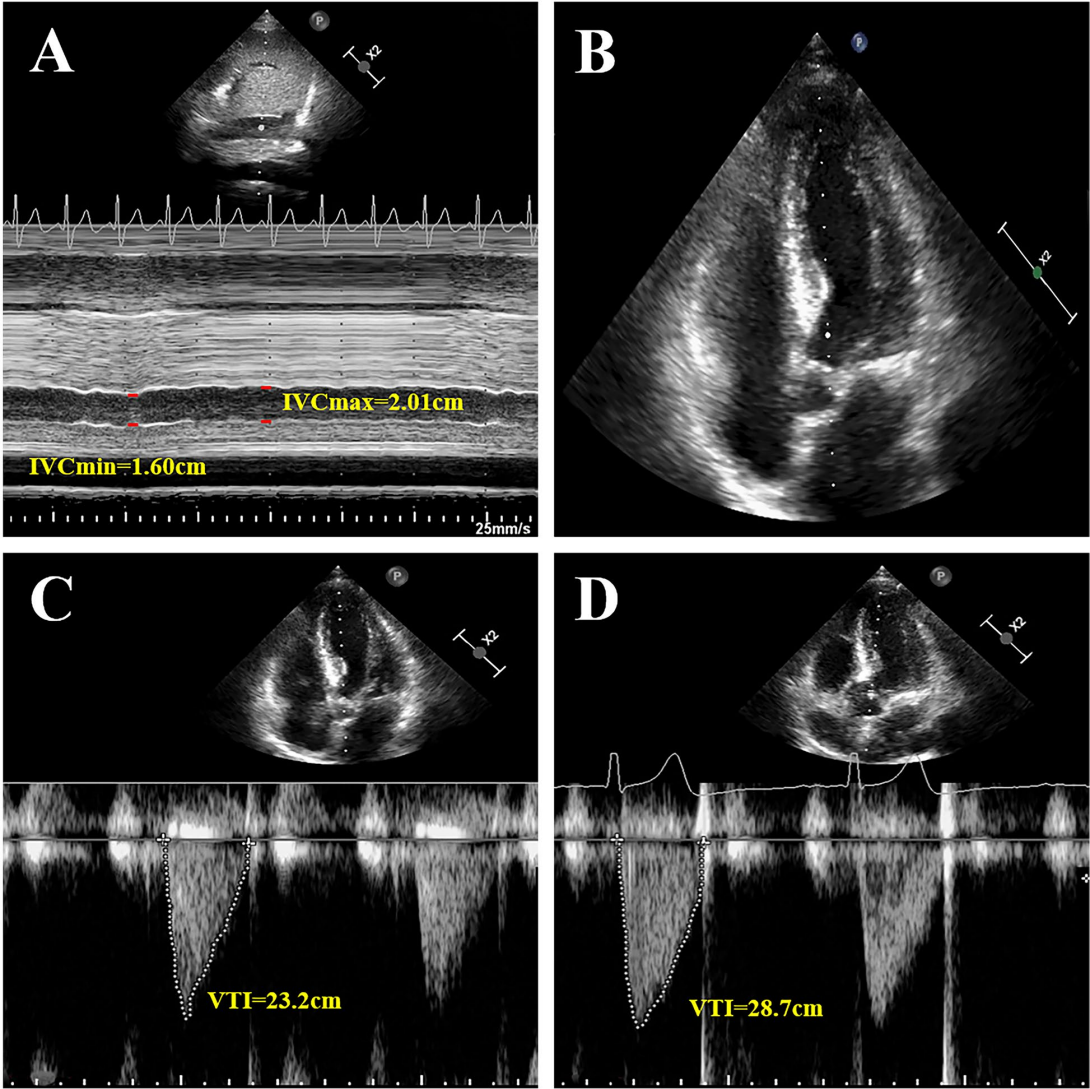
**A** shows the 2D image of the inferior vena cava and panel below shows the diameter of the inferior vena cava varying with respiration in M-mode. **B** shows the apical 5-chamber view. The sampling point is placed in the left ventricular outflow tract. **C** and **D** show the changes of velocity–time integral (VTI) in the left ventricular outflow tract before and after volume expansion, respectively. IVCmax: maximum diameter of IVC; IVCmin: minimum diameter of IVC

On arrival at the AICU immediately after the operation, the patients received positive pressure mechanical ventilation with the following parameters: tidal volume of 8 ml/kg, the inspiratory/expiratory ratio of 1:2, and positive end-expiratory pressure of 5 cmH_2_O before IVC ultrasound assessment. The phased-array ultrasound probe (8–10 Hz) was placed at the intersection between the right mid-axillary line and the fifth to seventh intercostal space of patients at a supine position with the probe marker towards cephalad. The maximum IVC diameter (IVCmax) on inspiration and IVCmin on expiration were measured under M-mode modality respectively. The diameter variation of IVC (cIVC1) was calculated using the equation: cIVC1 = (IVCmax-IVCmin)/IVCmax × 100%. Thereafter, the left ventricular outflow tract (LVOT) acquisition in a parasternal long-axis view and its bloodstream via pulse-doppler from five-chamber apical view yields LVOT velocity time integral (VTI) and CO (Fig. 1). All echocardiographic measurements were performed at least three times and averaged. The compound sodium chloride of 5 ml/kg was infused within 15 min, and the variables above were measured again. The measurement of respiratory variation of IVC and fluid challenge test were performed again when patients were extubated with spontaneous peaceful breathing. The collapsibility of IVC (cIVC) was calculated with following the equation: cIVC2 = (IVCmax-IVCmin)/IVCmax × 100%. The patients were assigned to the responder group (R) and non-responder group (NR) according to the augment of CO of more or less than 15% above the baseline before the fluid challenge test.

### Data collection

The indexes of heart rate (HR), mean arterial pressure (MAP), CVP, cIVC1 or cIVC2, the difference of the maximum and minimum IVC diameter (ΔIVC), VTI, cardiac index (CI) and CO of patients were collected at the following time-points: 5 min stabilization after patient entering AICU under mechanical ventilation (T1), following the first fluid challenge test (T2), 5 min stabilization after extubation under spontaneously breathing (T3) and following the second fluid challenge test (T4). The overall perioperative loss and supplementary volume in patients were recorded.

### Statistical analysis

The results of our pilot study of 20 patients showed that the AUC of cIVC1 predicted volume responsiveness under mechanical ventilation was 0.74, and the AUC of cIVC2 under spontaneous breathing was 0.8. Therefore, we choose a rather low value of 0.74 to calculate the sample size. The result showed that at least 54 patients were required to detect the difference of 0.24 between the AUC of cIVC1 (0.74) and the null hypothesis (0.5), with a power of 0.9 and a two-tailed type I error of 0.05, assuming the volume responsiveness incidence of 50% in patients after abdominal surgery. To allow for a possible 10% dropout rate, a sample size of 60 was used.

According to the Shapiro–Wilk test results, continuous data were presented as mean ± standard deviation or median (25 to 75% interquartile range) to test the normality of data distribution. Baseline hemodynamic parameters between the two groups were assessed using an independent Student’s t-test or Man-Whitney U test. The paired Student’s t-test or nonparametric Wilcoxon test was used to comparing the hemodynamic and echocardiographic indicators before and after the fluid challenge. Categorical data were compared using the χ2 test. According to the normality of two variables, the Pearson or Spearman correlation coefficient was used to assess the relationships between the percentage change in CO and IVC related parameters. Receiver operating characteristic (ROC) curve analysis was performed to test the predictive value of IVC related parameters, and the areas under the curve with 95% confidence interval (CI) were calculated. The best cut-off value was determined by the maximum of the Youden index (sensitivity + specificity-1). Comparison of AUCs between two indicators was performed using the methodology proposed by Delong and his colleagues [13]. The area between 90% sensitivity and 90% specificity was defined as a gray zone, which provided an uncertain range in which patients’ volume status was difficult to distinguish. Taking the clinical experience, relevant literature reviewed, and the model's stability into consideration, the following variables were included: MAP, HR, CVP, IVC-related parameters. Software SPSS (version 23.0, USA) and Medcalc 19.6.1 (version 19.6.1, Belgium) were employed for statistical analysis. A P-value less than 0.05 was considered statistically significant.

## Results

### Demographic and clinical characteristics

**Fig. 2:**
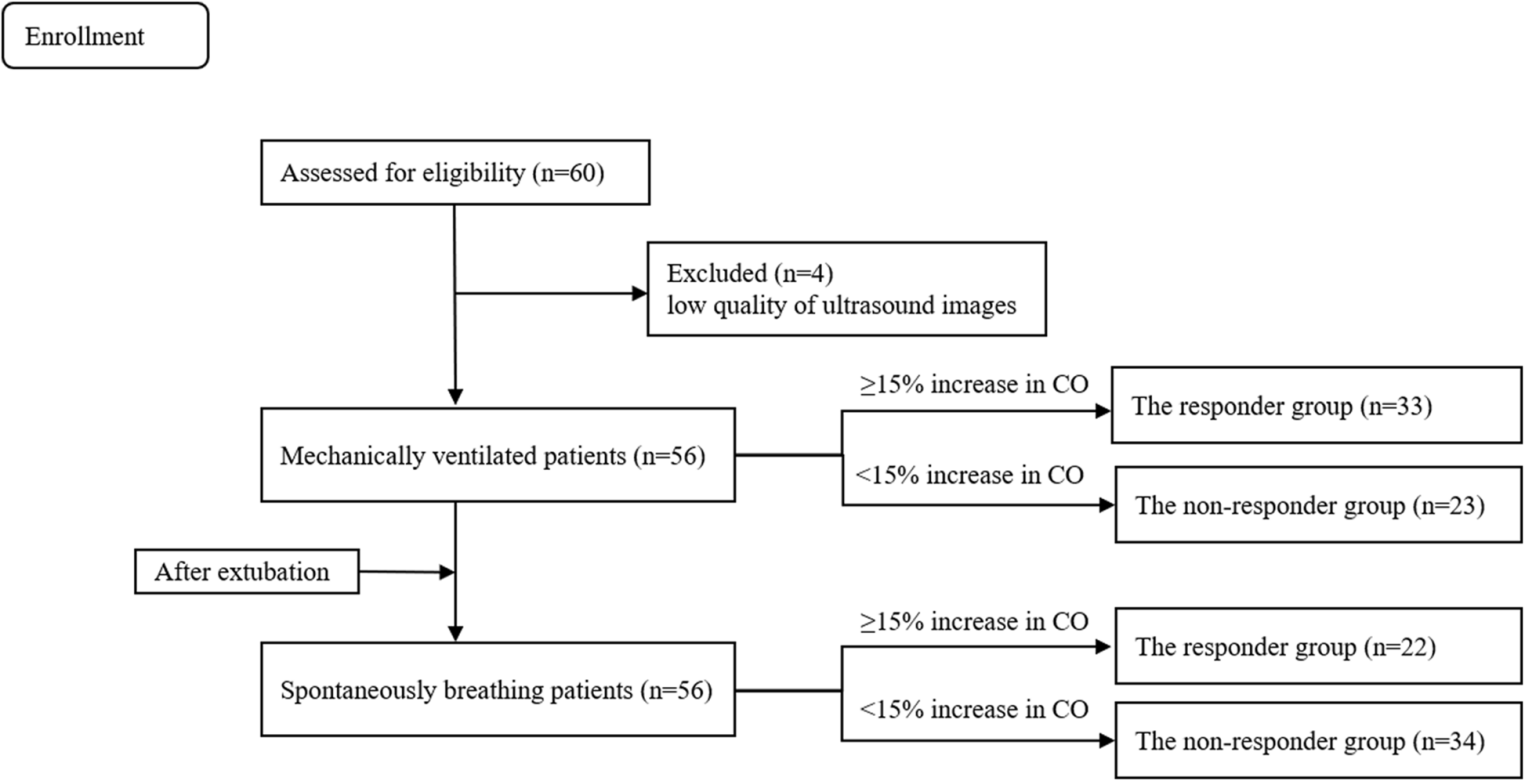
Flow diagram of patients. CO, cardiac output

A total of sixty patients were recruited in this study, and four patients were excluded due to low quality of ultrasound images. Thus, fifty-six patients completed the present clinical study. Thirty-three mechanically ventilated patients (58.9%) and twenty-two spontaneously breathing patients after the tracheal extubation (39.3%) responded to fluid challenge (Fig. 2). Demographic and clinical characteristics were comparable between the study groups under two respiratory modes (Table 1, *P* > 0.05).

**Table 1:** The demographic and clinical characteristics of patients

Respiration mode	Group	N	Sex (Male/Female)	Age (yr)	BSA (m^2^)	ASA (II/III)	Type of surgery (G/H/P)
Mechanical ventilation	R	33	21/12	56 ± 8	1.64 ± 0.17	17/16	17/12/4
	NR	23	10/13	58 ± 8	1.59 ± 0.20	12/11	12/8/3
Spontaneous breathing	R	22	11/11	58 ± 9	1.65 ± 0.13	10/12	10/8/4
	NR	34	20/14	57 ± 8	1.61 ± 0.21	19/15	19/12/3

There was no statistical difference in basic data between the two groups in both mechanically ventilation and spontaneously breathing patients (*p* > 0.05)

*BSA* body surface area, *ASA* American Society of Anesthesiologists physical status, *G/H/P* Gastrointestinal/Hepatobiliary/Pancreatic surgery

### In mechanically ventilated patients

#### Hemodynamic and echocardiographic measurements

At baseline, fluid responders had a smaller IVC diameter and a larger ΔIVC and cIVC1 than non-responders. After the volume expansion of 5 ml/kg in 15 min, the VTI, CO, and CI significantly increased in both groups (*P* < 0.05). Meanwhile, the fluid challenge significantly escalated IVCmax and IVCmin in group R and HR in group NR (*P* < 0.05, Table 2).

**Table 2:** Comparison of hemodynamic and echocardiographic parameters between two groups before and after fluid challenge test in mechanically ventilation patients

Parameters	Group R (*n* = 33)		Group NR (*n* = 23)	
	T1	T2	T1	T2
IVCmax (cm)	1.56 ± 0.31*	1.60 ± 0.33	1.74 ± 0.34	1.84 ± 0.32†
IVCmin (cm)	1.20 ± 0.29*	1.24 ± 0.34	1.46 ± 0.36	1.54 ± 0.34†
ΔIVC (cm)	0.35 (0.31–0.41)*	0.35 ± 0.12	0.27 (0.21–0.31)	0.30 ± 0.11
cIVC1 (%)	24.02 ± 5.93*	21.51 (17.68–26.89)	15.12 (12.57–19.46)	16.69 ± 6.65
CVP (cmH_2_O)	5 (2–6)	5 ± 3	5 ± 3	5 ± 3
MAP (mmHg)	95 ± 12	96 ± 11	95 ± 12	94 ± 11
HR (beats min^−1^)	60 (54–67)	64 ± 8†	62 (55–67)	61 (56–67)
VTI (cm)	24.5 ± 3.4	27.6 ± 4.3†	26.4 ± 4.0	27.7 ± 4.0†
CO (L min^−1^)	4.54 ± 0.98	5.41 ± 1.18†	5.10 ± 1.19	5.38 ± 1.26†
CI (L min^−1^ m^−2^)	2.78 ± 0.57*	3.31 ± 0.69†	3.23 ± 0.82	3.41 ± 0.85†

*T1* 5 min stabilization after patient entering AICU under mechanical ventilation, *T2* following the first fluid challenge test, *IVC* inferior vena cava, *IVCmax* maximum diameter of IVC, *IVCmin* minimum diameter of IVC, *ΔIVC* the difference of the maximum and minimum IVC diameter, *cIVC1* collapsibility of IVC in mechanically ventilated patients, *HR* heart rate, *VTI* velocity time integral, *CI* cardiac index, *CVP* central venous pressure, *MAP* mean arterial pressure, *CO* cardiac output

^*^ *P* < 0.05 versus group NR

^†^ *P* < 0.05 versus before fluid challenge test

**Fig. 3:**
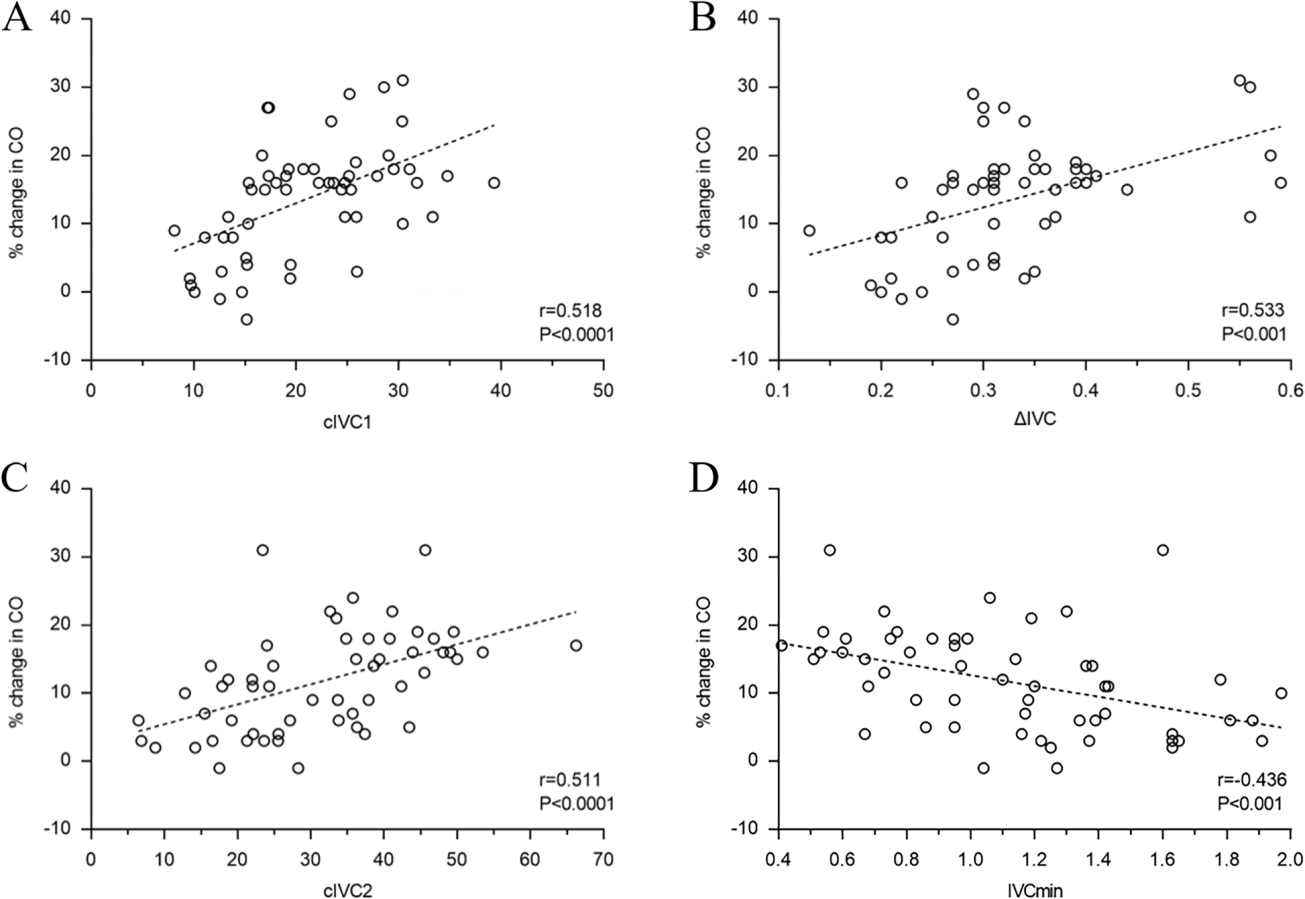
Relationships of the percentage change in CO with baseline cIVC1 (**A**) or ΔIVC (**B**) in mechanically ventilated patients and baseline cIVC2 (**C**) or IVCmin (**D**) in spontaneously breathing patients. Trend lines are presented as dotted lines. CO: cardiac output; IVC: inferior vena cava; cIVC1: IVC diameter variation in mechanically ventilated patients; ΔIVC: the difference of the maximum and minimum IVC diameter; cIVC2: collapsibility of IVC in spontaneously breathing patients; IVCmin: minimum diameter of IVC

The cIVC1 and Δ IVC at baseline closely correlated with the percentage change in CO after fluid challenge (r = 0.518, *P* < 0.0001; r = 0.533, *P* < 0.001; Fig. 3).

### Prediction of volume responsiveness

**Fig. 4:**
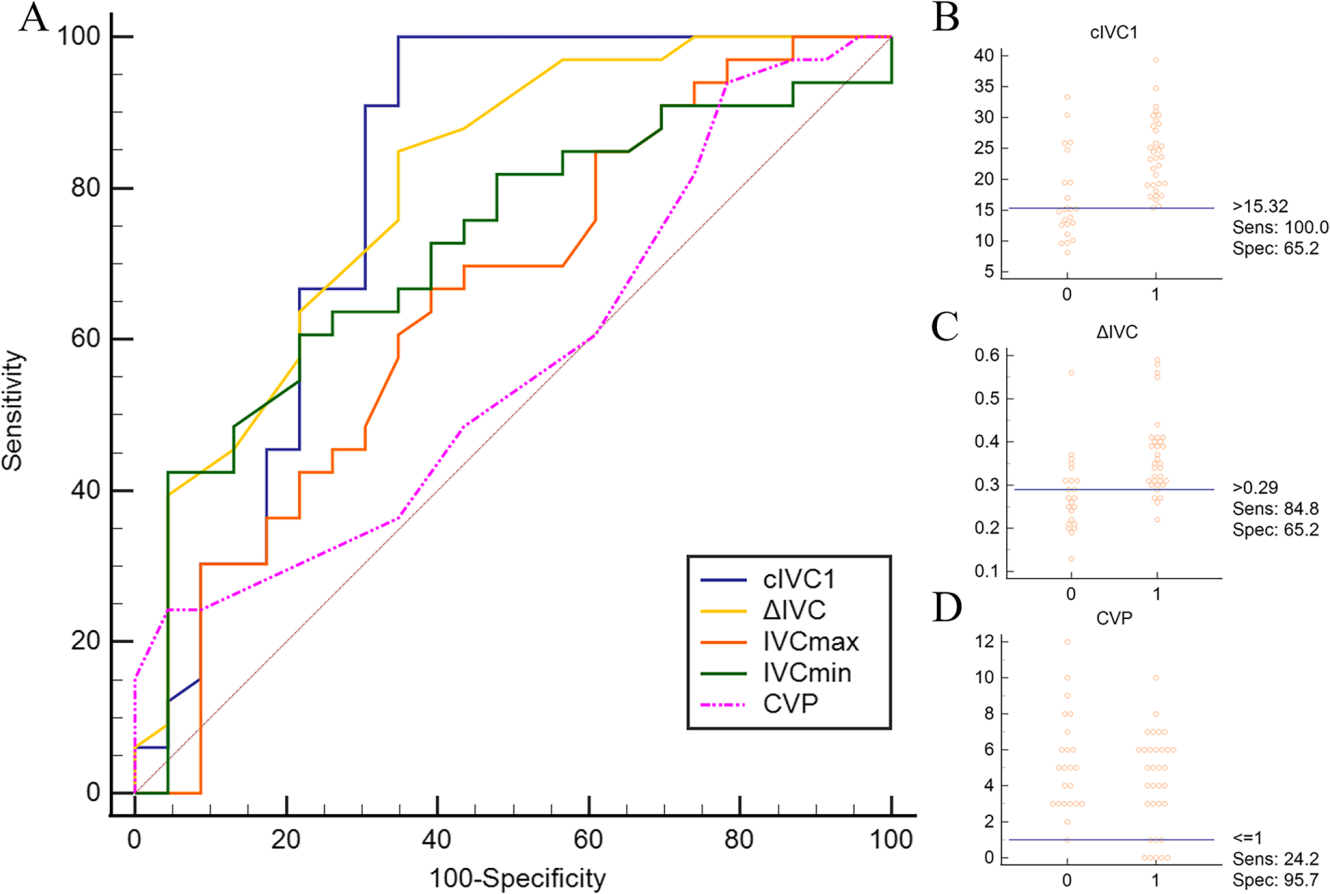
Receiver operating characteristic curves of IVC-related parameters and CVP (**A**) achieved from mechanically ventilated patients to predict volume responsiveness. Interactive dot diagram of cIVC1 (**B**), ΔIVC (**C**), CVP (**D**) showing the optimal cut-off value predicting volume responsiveness. IVC: inferior vena cava; cIVC1: IVC diameter variation in mechanically ventilated patients; ΔIVC: the difference of the maximum and minimum IVC diameter; IVCmax: maximum diameter of IVC; IVCmin: minimum diameter of IVC; CVP: central venous pressure; Sens: sensitivity; Spec: specificity

The AUC of cIVC1 or ΔIVC were 0.80 (95% CI: 0.68–0.90, *P* < 0.0001) and 0.80 (95% CI 0.67–0.90, *P* < 0.0001), respectively, which was not statistically different (*P* > 0.05), and were higher than that of CVP (*P* = 0.025, *P* = 0.021). The optimal cut-off value of cIVC1 to predict volume responsiveness was 15.32% with the sensitivity of 100% and specificity of 65%, respectively. The ΔIVC had the same specificity compared with cIVC1 when the cut-off value was 0.29 cm but a lower sensitivity of 85% (Table 3, Fig. 4).

**Table 3:** Prediction of fluid responsiveness using the receiver operating characteristic curves and gray zones of IVC related parameters and CVP under two respiratory modes

Respiratory mode	Parameters	AUROC curve (95% CI)	*p*-value	Optimal cut-off value	Sensitivity (%)(95% CI)	Specificity (%)(95% CI)	Youden index	Gray zones	Patients in gray zones (%)
Mechanical ventilation	cIVC1 ^*^	0.80 (0.68–0.90)	< 0.0001	> 15.32	100 (89–100)	65 (43–84)	0.65	17.01–25.93	44.6
	ΔIVC ^*^	0.80 (0.67–0.90)	< 0.0001	> 0.29	85 (68–95)	65 (43–84)	0.50	0.27–0.36	48.2
	IVCmax	0.65 (0.51–0.77)	> 0.05	≤ 1.69	67 (48–82)	61 (39–80)	0.28	1.32–1.96	60.7
	IVCmin	0.73 (0.60–0.84)	0.002	≤ 1.44	88 (72–97)	57 (35–77)	0.44	0.99–1.54	57.1
	CVP	0.57 (0.43–0.70)	> 0.05	≤ 1	24 (11–42)	96 (78–100)	0.20	2.04–9.69	76.8
Spontaneous breathing	cIVC2 ^*^,†	0.87 (0.75–0.94)	< 0.0001	> 30.25	91 (71–99)	71 (53–85)	0.62	30.72–38.32	23.2
	ΔIVC	0.76 (0.62–0.86)	< 0.001	> 0.46	86 (65–97)	62 (44–78)	0.48	0.41–0.61	51.8
	IVCmax	0.77 (0.64–0.87)	< 0.0001	≤ 1.53	73 (50–89)	71 (53–85)	0.43	1.29–1.87	51.8
	IVCmin ^*^	0.85 (0.73–0.93)	< 0.0001	≤ 1.14	86 (65–97)	71 (53–85)	0.57	0.82–1.19	30.4
	CVP	0.64 (0.50–0.76)	> 0.05	≤ 4	82 (60–95)	47 (30–65)	0.29	0–4.9	64.3

*AUROC* area under the receiver operating characteristic, *IVC* inferior vena cava, *cIVC1* IVC diameter variation in mechanically ventilated patients, *ΔIVC* the difference of the maximum and minimum IVC diameter, *IVCmax* maximum diameter of IVC, *IVCmin* minimum diameter of IVC, *cIVC2*: collapsibility of IVC in spontaneously breathing patients

^*^ cIVC1 versus CVP, *P* = 0.025; ΔIVC versus CVP, *P* = 0.021; cIVC2 versus CVP, *P* = 0.0039; IVCmin versus CVP, *P* = 0.0053

^†^ cIVC versus ΔIVC, *P* = 0.037

### In spontaneously breathing patients

#### Hemodynamic and echocardiographic measurements

As is presented in Table 4, patients in group R had a smaller IVC diameter and a larger ΔIVC and cIVC2 at baseline. Compared with the parameters before the fluid challenge, HR, VTI, CO, and CI increased in both groups. The same situation happened in ΔIVC and cIVC in responders.

**Table 4:** Comparison of hemodynamic and echocardiographic parameters between two groups before and after fluid challenge test in spontaneously breathing patients

Parameters	Group R (*n* = 22)		Group NR (*n* = 34)	
	T3	T4	T3	T4
IVCmax (cm)	1.30 (1.07–1.58)*	1.34 ± 0.23	1.72 ± 0.33	1.76 ± 0.27
IVCmin (cm)	0.82 ± 0.30*	0.78 (0.69–0.95)	1.30 ± 0.36	1.34 ± 0.34
ΔIVC (cm)	0.55 ± 0.12*	0.45 (0.37,0.59)†	0.42 ± 0.16	0.41 ± 0.13
cIVC2 (%)	41.45 ± 9.75*	36.15 ± 10.13†	25.08 ± 10.54	24.48 ± 9.12
CVP (cmH_2_O)	3 (0–4)	2 (0–4)	4 (1–5)	3 (1–6)
MAP (mmHg)	92 ± 14	96 (83–104)	91 ± 10	92 ± 10
HR (beats min^−1^)	69 (62–73)	70 ± 11†	70 ± 7	72 ± 7†
VTI (cm)	24.8 ± 3.1	28.3 ± 4.4†	25.5 ± 3.7	26.5 ± 4.0†
CO (L min^−1^)	5.08 ± 1.02	6.06 ± 1.26†	5.36 ± 1.05	5.73 ± 1.15†
CI (L min^−1^ m^−2^)	3.09 ± 0.62	3.68 ± 0.76†	3.38 ± 0.76	3.62 ± 0.84†

*T3* 5 min stabilization after extubation under spontaneously breathing, *T4* following the second fluid challenge test, *IVC* inferior vena cava, *IVCmax* maximum diameter of IVC, *IVCmin* minimum diameter of IVC, *ΔIVC* the difference of the maximum and minimum IVC diameter, *cIVC2* collapsibility of IVC in spontaneously breathing patients, *HR* heart rate, *VTI* velocity time integral, *CI* cardiac index, *CVP* central venous pressure, *MAP* mean arterial pressure, *CO* cardiac output

^*^ *P* < 0.05 versus group NR

^†^ *P* < 0.05 versus before fluid challenge test

Baseline cIVC2 and IVCmin correlated significantly with the percentage change in CO after fluid challenge (*r* = 0.511, *P *< 0.0001; *r* = -0.436, *P* < 0.001; Fig. 3). There was a weak correlation between the percentage change in CO and baseline IVCmax, ΔIVC and CVP (*r* = -0.36, *P* = 0.006; *r* = 0.39, *P* = 0.003; *r* = -0.334,* P* = 0.012).

### Prediction of volume responsiveness

**Fig. 5:**
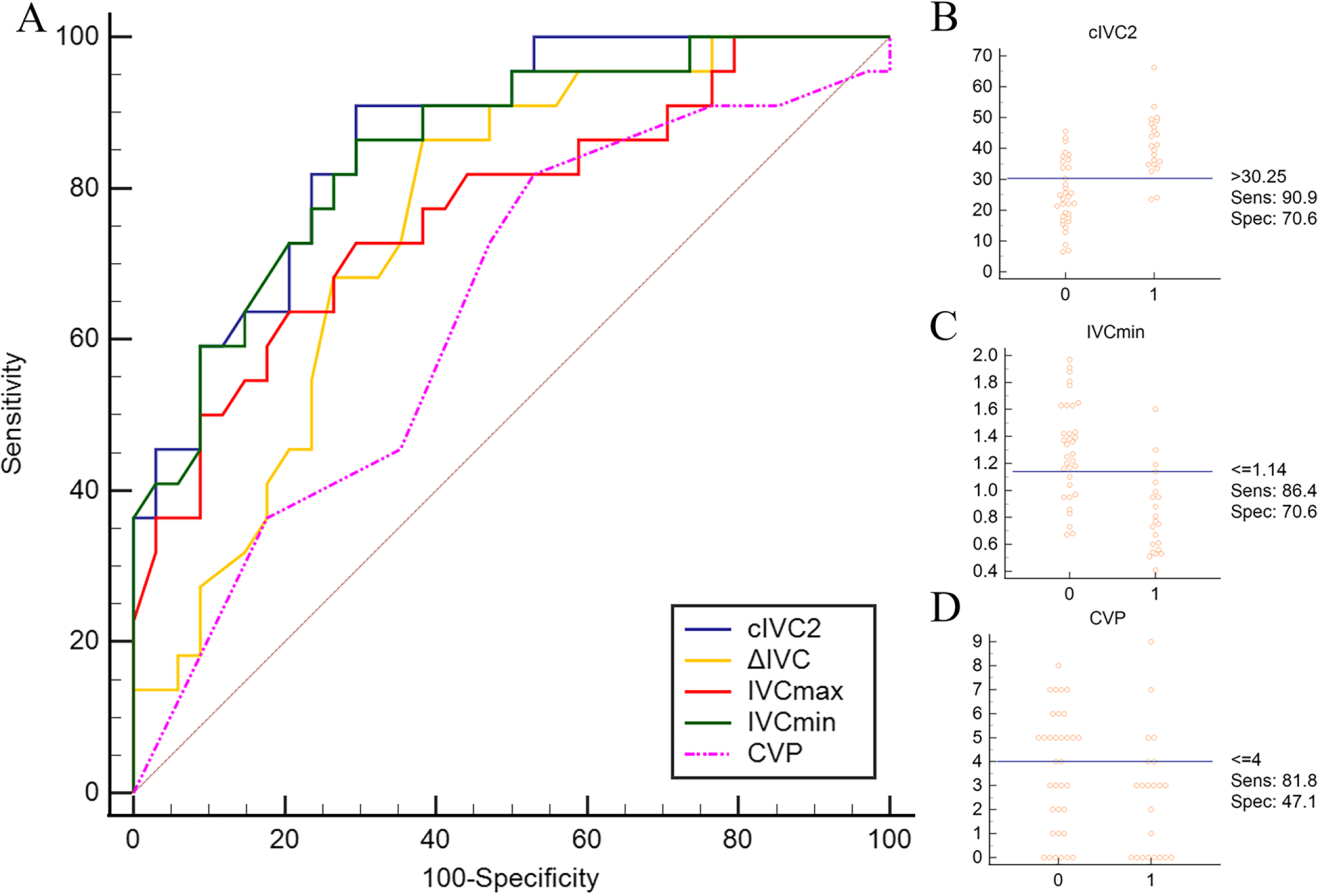
Receiver operating characteristic curves of IVC-related parameters and CVP (**A**) obtained in spontaneously breathing patients to predict volume responsiveness. Interactive dot diagram of cIVC2 (**B**), IVCmin (**C**), CVP (**D**) in spontaneously breathing patients showing the optimal cut-off value predicting volume responsiveness. IVC: inferior vena cava; cIVC2: collapsibility of IVC in spontaneously breathing patients; ΔIVC: the difference of the maximum and minimum IVC diameter; IVCmax: maximum diameter of IVC; IVCmin: minimum diameter of IVC; CVP: central venous pressure; Sens: sensitivity; Spec: specificity

A cIVC2 of 30.25% distinguished responders from non-responders with a 91% sensitivity and specificity of 71%, respectively. The AUC of cIVC2 was 0.87 (95% CI: 0.75–0.94) which was higher than 0.76 (95% CI: 0.62–0.86) of ΔIVC (*P* = 0.037). It is worth noting that there is no statistical difference in the AUCs between static indicator IVCmin and cIVC2, presenting similar diagnostic accuracy. The optimal cut-off value of IVCmin was 1.14 cm, with 86% sensitivity and specificity of 71%. The AUCs of both cIVC2 and IVCmin were statistically higher than CVP (*P* = 0.0039, *P *= 0.0053, Table 3, Fig. 5).

### Comparing the gray zones of IVC-related parameters in predicting volume responsiveness in patients under two respiratory modes

**Fig. 6:**
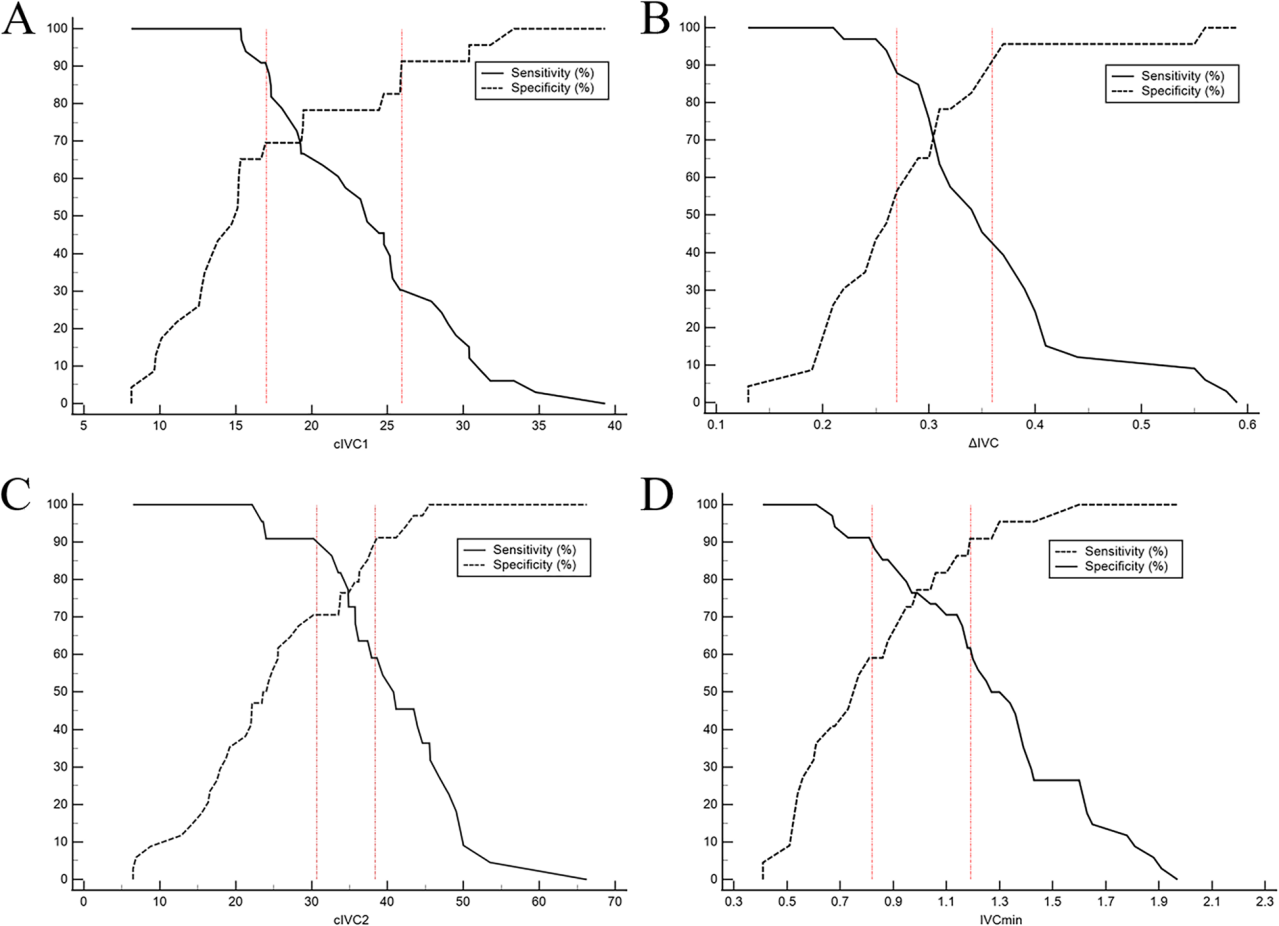
Sensitivity and specificity plots and gray zones of cIVC1 (**A**) and ΔIVC (**B**) in mechanically ventilated patients and cIVC2 (**C**) and IVCmin (**D**) in spontaneously breathing patients to reflect the ability of predicting volume responsiveness. The two dotted lines indicate the gray zone. IVC: inferior vena cava; cIVC1: IVC diameter variation in mechanically ventilated patients; ΔIVC: the difference of the maximum and minimum IVC diameter; cIVC2: collapsibility of IVC in spontaneously breathing patients; IVCmin: minimum diameter of IVC

To avoid the dualism of a single cut-off value, the gray zone bounded by a sensitivity of 90% and a specificity of 90% provided an uncertain range. The gray zone for cIVC2 was 30.72 to 38.32% and included 23.2% of spontaneously breathing patients. By contrast, it was 17.01 to 25.93% for cIVC1 and included 44.6% of mechanically ventilated patients. The proportions of subjects in the gray zone for inferior vena cava respiratory variation under two respiratory modes were the lowest among all IVC relevant parameters. The number of spontaneously breathing patients in the gray zone for IVCmin was 17 (30.4%), which was lower than 29 (51.8%) for ΔIVC, indicating that IVCmin had a better test utility (Table 3; Fig. 6).

### The independent predictors analyzed by multivariable logistic regression under two respiratory modes

After adjusting some indicators commonly applied in the clinical setting, the multivariable logistic regression analysis results indicated that inferior vena cava respiratory variation was an independent predictor of volume responsiveness in mechanically ventilated patients and spontaneously breathing patients. A larger cIVC in mechanically ventilated or spontaneously breathing modes showed a higher probability in responding to volume expansion, with the odds ratio of 1.34 (95% CI: 1.12–1.62) and 1.16 (95% CI: 1.05–1.28), respectively (Supplementary Table S1 and S2).

## Discussion

Our results indicated that the IVC respiratory variability by ultrasound measurement exhibited a close relationship with the percentage change in CO after fluid challenge. Furthermore, IVC respiration variability were moderately predictive of volume responsiveness in postoperative patients either in mechanically ventilated or spontaneously breathing mode. This study's intriguing finding was the cIVC and IVCmin were the optimal predictor of volume responsiveness for spontaneously breathing patients with few patients in the gray zone, an area of overlapping between responders non-responders, and challenging to evaluate in a dichotomous manner.

Among dynamic parameters, the predictor of IVC respiratory variation has higher reliability than SVV or PPV in assessing volume responsiveness in mechanically ventilated patients [14]. Consistent with previous studies, the AUC of cIVC1 was as high as 0.80 in the present study [15]. The cut-off value of cIVC1 of 15.32% predicted the volume responsiveness in postoperative patients with a sensitivity of 100%, specificity of 65%, respectively. In line with the previous findings, the threshold of cIVC1 showed a different accuracy, particularly a lower specificity in surgical patients [16]. Therefore, we still had a higher probability of making a “false positive” error; that is, 35% of non-responders under mechanic ventilation with cIVC1 over 15.32% were evaluated as “volume responder” with a mistake. Furthermore, ΔIVC was also efficacious in predicting the volume status in patients as cIVC1, with the AUC of 0.80. Both parameters were closely correlated with the percentage change in CO following fluid challenge as opposed to the static parameters of IVCmax or IVCmin. As is well-known, the intrathoracic pressure changes during positive pressure ventilation result in the periodic changes of the venous return to the right atrium, which is closely related to the preload of right ventricles and ultimately influencing the SV and CO. In the steep limb of the Frank-Starling curve compared with the plateau portion, the same change in venous return due to intrathoracic pressure leads to a marked increase in SV, facilitating venous return and increasing the variance of the IVC in diameter over the respiratory phase, which accounts for the IVC variation index as one of the optimal candidates in volume assessment [17].

Data on the accuracy of IVC variations for predicting fluid needs in spontaneously breathing patients are scarce [18]. Besides the common confounders of venoplegia in sepsis, right ventricle failure, or severe tricuspid regurgitation, etc. influencing the volume assessment in spontaneously breathing patients and artificially ventilated patients as well, the inconstant forced inspiratory effort, the changed rhythm of respiration, or the varied intraabdominal pressure as unique characteristic in spontaneously breathing patients after surgery has a huge impact on venous return to the right atrium, thus affecting the diameter of IVC, which is independent of the volume responsiveness [12]. As re-emphasized by Muller and colleagues, it seems hazardous to manage fluids in a spontaneously breathing patient by using IVC respiratory variations only until further data are published [18]. However, the results from Corl [10]  showed that cIVC could predict the increase of CO after fluid infusion when cIVC was greater than 25% with a sensitivity of 87% and specificity of 81% in 124 critically ill patients with spontaneously breathing in ICU. Our results showed that the cut-off value of cIVC2 was 30.25% with high sensitivity of 91% and moderate specificity of 71%. Inconsistent with the studies conducted by Airapetain et al. [11], they found that cIVC > 42% can predict volume responsiveness accurately in spontaneously breathing patients. These findings reiterated that the caval index threshold to evaluate the volume status varied in different clinical settings. In the present study, cIVC2 and IVCmin were superior to cIVC1 in terms of few patients in the gray zone, suggesting that cIVC in spontaneously breathing patient was highly clinically useful. The reasons why IVC variation index is a better predictor of volume responsiveness in spontaneously breathing patients in this study can be explained as follows: our patients scheduled to undertake abdominal surgery were healthier in physical status of ASA with less comorbidities, and well-preserved cardiac function compared with the critically ill patients in ICU commonly complicated with hemodynamic instability. The patients in AICU received postoperative analgesic management after extubation, the shallow and rapid breathing pattern associated with incision pain can be avoided [19]. All patients in the present study have an uneventful recovery after surgery without intraabdominal pressure increase secondary to abdominal compartmental syndrome.

Relative to the IVC variation index above, the CVP was less reliable in predicting the volume responsiveness in patients regardless of respiratory status after abdominal surgery. Consistent with the previous studies, the AUC for CVP was below 75% [20]. The baseline CVP has a weak relationship with the percentage change in CO after fluid challenge, denoting little volume appraisal value. In addition, there are considerable factors, including the right heart systolic function, severe tricuspid regurgitation, pulmonary hypertension, respiratory stress, and increased pericardial cavity pressure that precluded CVP from assessing the volume status accurately [19].

Inevitably, there are some limitations in this study. Firstly, only patients scheduled to undergo abdominal operations were recruited. Whether the IVC variation index can be extrapolated to the other types of surgical is far from elucidated, therefore a multi-center prospective randomized clinical trials are warranted. Secondly, the cardiac ultrasonographic assessment were not reviewed by a professional sonographic physician, but multiple measurements strictly controlled the intra- or inter-observer variation for each parameter by two residents from the Department of Anesthesiology with proficiency in cardiac ultrasound examination. Thirdly, the gold standard for CO measurement is to place a pulmonary artery catheter (PAC) via thermal dilution method. However, it is unrealistic to take a high risk of placing PAC in patients undergoing abdominal surgery. We performed triple measurements for every single parameter, and the average of these values was adopted in the end to ensure the accuracy of CO measurement.

## Conclusions

The IVC respiratory variability index was moderately predictive of postoperative volume responsiveness of patients undergoing abdominal surgery under artificially or spontaneously breathing. The cIVC and IVCmin seemed to be more clinically useful in predicting the volume responsiveness in the spontaneously breathing patient as opposed to the predictor of cIVC in mechanically ventilated patients.

## Supplementary Information

**Additional file 1. **

**Additional file 2.** 

## Data Availability

All data generated or analyzed during this study are included in this published article.

## Abbreviations

AICU: Anesthesia intensive care unit
ASA: American Society of Anesthesiologists
AUC: Area under the receiver operating characteristic
BMI: Body mass index
CI: Confidence interval
CI: Cardiac index
cIVC1: IVC diameter variation in mechanically ventilated patients
cIVC2: Collapsibility of IVC in spontaneously breathing patients
CO: Cardiac output
CVP: Central venous pressure
HR: Heart rate
ICU: Intensive care unit
IVC: Inferior vena cava
IVCmin: Minimum IVC diameter
IVCmax: Maximum IVC diameter
ΔIVC: The difference of the maximum and minimum IVC diameter
LVOT: Left ventricle outflow tract
MAP: Mean arterial pressure
PPV: Pulse pressure variation
ROC: Receiver operating characteristic
SVV: Stroke volume variation
VTI: Velocity time integral
