# Supplementary material for: The diagnostic accuracy of inferior vena cava respiratory variation in predicting volume responsiveness in patients under different breathing status following abdominal surgery
Source: BMC Anesthesiol. 2022 Mar 8;22:63. doi: 10.1186/s12871-022-01598-5 (PMC8903007; doi:10.1186/s12871-022-01598-5)
Supplement: Supplementary file 1 — Additional file 1. [file 12871_2022_1598_MOESM1_ESM.docx]

| **Supplementary Table S1.** Multivariable logistic regression analysis for fluid responsiveness in postoperative mechanically ventilated patients | | | | |
| --- | --- | --- | --- | --- |
| Predictors | Regression Coefficient | Odds Ratio | 95%CI | P-value |
| MAP | -0.06 | 0.95 | 0.88-1.02 | 0.139 |
| HR | -0.09 | 0.92 | 0.82-1.02 | 0.092 |
| CVP | -0.15 | 0.86 | 0.66-1.13 | 0.295 |
| Net intraoperative infusion volume | -0.001 | 0.999 | 0.999-1 | 0.05 |
| IVCmax | -0.50 | 0.61 | 0.03-12.23 | 0.746 |
| cIVC1 | 0.296 | 1.34 | 1.12-1.62 | 0.002 |
| Constant | 9.35 | NA | NA | 0.11 |
| MAP: mean arterial pressure; HR: heart rate; CVP: central venous pressure; IVC: inferior vena cava; IVCmax: maximum diameter of IVC; cIVC1: collapsibility of IVC in mechanically ventilated patients; NA: not applicable. CI: confidence interval. | | | | |
