# Supplementary material for: The diagnostic accuracy of inferior vena cava respiratory variation in predicting volume responsiveness in patients under different breathing status following abdominal surgery
Source: BMC Anesthesiol. 2022 Mar 8;22:63. doi: 10.1186/s12871-022-01598-5 (PMC8903007; doi:10.1186/s12871-022-01598-5)
Supplement: Supplementary file 2 — Additional file 2. [file 12871_2022_1598_MOESM2_ESM.docx]

| **Supplementary Table S2.** Multivariable logistic regression analysis for fluid responsiveness in postoperative spontaneously breathing patients | | | | |
| --- | --- | --- | --- | --- |
| Predictors | Regression Coefficient | Odds Ratio | 95%CI | P-value |
| MAP | 0.18 | 1.02 | 0.95-1.09 | 0.611 |
| HR | -0.08 | 0.93 | 0.84-1.02 | 0.109 |
| CVP | 0.08 | 1.08 | 0.75-1.56 | 0.675 |
| IVCmax | -1.01 | 0.36 | 0.02-6.25 | 0.485 |
| cIVC2 | 0.15 | 1.16 | 1.05-1.28 | 0.003 |
| Constant | -0.44 | NA | NA | 0.931 |

MAP: mean arterial pressure; HR: heart rate; CVP: central venous pressure; IVC: inferior vena cava; IVCmax: maximum diameter of IVC; cIVC2: collapsibility of IVC in spontaneously breathing patients; NA: not applicable. CI: confidence interval.
